# Understanding the Mental and Physical Burdens of Physicians and Identifying Support Interventions in Bangladesh: Qualitative Study

**DOI:** 10.2196/76934

**Published:** 2025-09-26

**Authors:** Rahat Jahangir Rony, Shams Akbar Aalok, Lamia Amin Tisha, Marzan Mahatab, Nova Ahmed

**Affiliations:** 1 Department of Computer Science and Informatics School of Computer Science Cardiff University Cardiff United Kingdom; 2 Department of Electrical and Computer Science North South University Dhaka Bangladesh

**Keywords:** COVID-19 pandemic, Bangladeshi doctors, doctors’ mental burden, doctors’ physical burden, doctors’ well-being, doctors’ workload, doctors’ families health, well-being model, contextual support and social awareness, technology interventions, artificial intelligence, AI

## Abstract

**Background:**

The COVID-19 pandemic had a substantial, negative impact on the world, and physicians played a crucial role in providing health care while facing the risk of contracting the virus themselves. While working on the frontlines, they also needed to protect themselves and their families from the virus. Unfortunately, their mental health was not given the attention it deserved. Many physicians experienced burnout due to the numerous challenges they faced, yet they received little support. Resource-limited countries such as Bangladesh were particularly affected due to a lack of resources. Although high-income countries have proposed a well-being model for physicians, this model is not directly applicable to resource-limited nations. However, redefining the model to suit the specific needs of physicians in resource-limited countries could provide sustainable support for their well-being.

**Objective:**

We aimed to gain a deeper understanding of the mental and physical burdens faced by Bangladeshi physicians during the COVID-19 pandemic, and the contextual factors influencing their well-being. By understanding these aspects, we can recommend an adaptable, effective, and sustainable contextual model.

**Methods:**

We conducted semistructured online interviews with 14 physicians in Chattogram, Bangladesh, during the COVID-19 pandemic. The physicians actively working in the COVID-19 unit were recruited from public and private hospitals through purposive sampling. Participants were aged between 25 and 35 years and had up to 8 years of working experience, including 43% (6/14) interns, 36% (5/14) medical officers, 14% (2/14) researchers, and 7% (1/14) surgeons. Each interview was conducted in Bengali, and we obtained consent to record the audio. Overall, 637 minutes of discussion were translated and transcribed. The results were analyzed using reflexive thematic analysis.

**Results:**

We identified factors that impacted physicians’ mental and physical health and well-being during the COVID-19 pandemic. They frequently dealt with undiagnosed patients, which put them at risk. Physicians often feared the potential danger their profession posed to their families, choosing to prioritize their family’s safety over their own. In addition, heavy workloads, excessive duty hours, and a shortage of colleagues substantially affected their sleep patterns and disrupted their regular work schedules. Instead of receiving societal support, they often faced negative perceptions from the public. In addition, during times of mass patient deaths, many physicians struggled to cope with their emotions without any mental health support.

**Conclusions:**

Our work shows physicians’ mental and physical health burdens with various contextual difficulties. We understood these concerns and suggested a contextual (emphasizes understanding and addressing users’ behavior within its specific context) intervention model inspired by the well-being framework. We emphasize the importance of integrating both contextual and technological interventions. Through this model, our goal is to involve stakeholders in redesigning the work environment for physicians, ensuring it is sustainable in the long term and adaptable to different situations.

## Introduction

### Background

The COVID-19 pandemic has led to a significant loss of human life globally [1-5], with physicians serving as frontline warriors dedicated to the well-being of the population while trying to keep themselves and their families safe [6,7]. Tragically, many physicians lost their lives while performing their professional duties. In the United States, over 4500 physicians succumbed to SARS-CoV-2 [8], while approximately 6500 health care professionals died in England and Wales in the United Kingdom [9]. These tragic figures were not isolated to high-income nations. The crisis extended to India, with approximately 1800 deaths of health care workers [10], and China reported the loss of 64 health care workers [11], among many other countries. The situation was similarly dire in Bangladesh, a resource-limited country, which reported around 113 physicians’ deaths within just a few months in 2020 [12]. With about 80,000 physicians caring [12] for a population of 180 million in Bangladesh, the high patient-to-physician ratio highlighted the immense pressure physicians face. This pandemic placed additional strain on physicians and the health care system, resulting in significant psychological distress [13].

While numerous studies have discussed the health effects of COVID-19 on patients, the impact on physicians has received less attention. Physician burnout became a prominent worldwide issue during the pandemic [14-16]. This burnout stemmed from several factors, including stress [17,18], workload [19,20], anxiety and depression [18,21], inadequate support [17,18,20], lack of protective gear [13,20], and many more. Research indicated that 32.9% of physicians experienced stress, 34.9% dealt with depression, and 39.5% faced anxiety [22]. In addition, a study by Emre et al [23] found that among 225 physicians, 17.8% exhibited symptoms of posttraumatic stress disorder, regardless of their gender, marital status, and other factors.

Factors contributing to this distress included a high risk of infection, inadequate protection from exposure, excessive workload, frustration, discrimination, isolation, negative emotions from patients, lack of family contact, and overall exhaustion [24,25]. Physicians often had to maintain social distance from their families to reduce the risk of transmitting the virus, further impacting their mental health [13,26]. In addition, during the pandemic, the extreme workload due to a surge in patients resulted in increased stress and depression [20,21]. Covering for colleagues [19,27] is a duty hour complexity [19] that also contributed to the increased workload. Because of these high work demands (mental, emotional, physical, and temporal), limited recovery experiences (such as relaxation and detachment from work) impact physicians’ mental health [18]. Many physicians also experienced sleep disturbances, with more than two-thirds reporting insomnia (68.3%) and a majority (93.7%) experiencing stress as a result [15]. Despite risking their lives serving patients, physicians received inadequate support from hospitals, government agencies, and society at large [20,28]. A lack of logistics support, insufficient backing from peers and supervisors, and low feelings of occupational competence during COVID-19–related tasks contributed to the emotional toll on physicians [18].

Much of the existing research has focused on physicians’ experiences in high-income countries, overlooking the burdens and well-being of physicians in resource-limited nations. Although some issues are similar, conditions are often worse in resource-limited countries, which struggle significantly with resource shortages and inadequate administrative support [20,29,30]. The pandemic has exacerbated the existing shortcomings within these health care systems, creating additional challenges for physicians. Therefore, there is an urgent need to explore the mental and physical health of physicians in the context of resource-limited countries and the unique problems they face.

### Aims and Approach

This research aimed to understand the mental and physical burdens faced by Bangladeshi physicians during the COVID-19 pandemic, considering the specific contextual challenges they encountered. We sought to explore how they delivered care to patients while also managing their personal and professional lives, the sociocontextual problems they faced during the pandemic, and how these issues impacted their overall well-being. However, there is limited research on how physicians can sustain their well-being. Daniels et al [31] presented a well-being model for health care professionals during the pandemic, which identified several key factors necessary for maintaining well-being, including access to basic physical resources, communication, embedded support systems, and psychological interventions. This model inspired us, and a similar approach tailored to address the specific concerns and contextual problems faced by physicians in Bangladesh would enhance its effectiveness and sustainability. In this paper, we report the results of in-depth interviews conducted with physicians in Bangladesh.

## Methods

We conducted in-depth qualitative interviews with 14 physicians in Bangladesh during the COVID-19 pandemic to understand their well-being and stress indicators. The interviews were semistructured and held online.

### Research Settings and Self-Disclosure

All participants were located in Chattogram, Bangladesh, which is the country’s largest port city and second-major city, situated approximately 300 km southeast of the capital, Dhaka. During the pandemic, Chattogram experienced a high frequency of COVID-19 infections, prompting us to recruit physicians from this area.

It is important for the readers to understand the researchers’ perspectives [32]. We chose this location because we are familiar with it. All researchers (2 men and 3 women) were born and raised in Bangladesh, making them well-acquainted with the local cultural norms. As native speakers, the researchers have conducted multidisciplinary research related to health and technology. These experiences allowed us to effectively facilitate this study.

### Recruitment and Participants

We focused on recruiting physicians who worked in both public and private hospitals during the COVID-19 pandemic. We used a purposive sampling method [33] to recruit 14 participants to accomplish this. During recruitment, we used close connections, such as relatives, friends, and colleagues, to identify potential participants. We explained our inclusion criteria (physicians who worked as frontliners in hospitals during the intense period of the pandemic) to our contacts, who helped us connect with eligible individuals. Then, we contacted them by phone to explain our research objectives and interview techniques. Subsequently, we formally recruited interested participants.

The 14 recruited participants were aged between 25 and 35 years. They had between 1 and 8 years of work experience in public and private hospitals in Bangladesh. In total, 12 (86%) participants were unmarried, while the remaining 2 (14%) were married. The group included 6 (43%) intern physicians (who had recently completed their professional degrees), 5 (36%) medical officers (serving as junior physicians), 2 (14%) researchers (focused on medical topics), and 1 (7%) surgeon (a senior physician). A detailed description of the participants has been discussed in the Results section.

Although the sample size was small, we reached a saturation level within that sample because we conducted in-depth interviews. After each interview, we organized and analyzed the data to improve our approach for the next one. Regardless of the participants’ gender, job positions, workplace, or years of experience, we found that they shared similar experiences during their duties throughout the pandemic, confirming the saturation of data.

### Ethical Considerations

This research was approved by the ethics committee at North South University, Bangladesh (2021/OR-NSU/IRB/0303). Before the interviews, we provided participants with soft copies of consent forms that included the research objectives, aims, and methods in Bengali. We clearly explained that the interviews would be audio recorded and that we would collect demographic information, such as name, age, gender, designation, and workplace. We assured participants that all information would be anonymized to protect their identities. They returned the consent forms online to confirm their participation before the interviews began.

We avoided using participants’ names, home addresses, and institutional affiliations as identifiers to maintain confidentiality. Instead, participants were assigned code names, such as participant 1, participant 2, ..., and participant 14. They were also informed that they had the complete right to withdraw from the interview at any time without losing their incentives. Only the researchers had access to the data, which was securely stored on a locked drive. These data will not be shared or transferred to any third-party entities. After the interviews, all participants received notebooks and stationery items sent via courier as a token of appreciation for their participation.

### Procedure and Study Design

We conducted interviews between May and December 2021. The participants had busy schedules due to their duty hours; in addition, to maintain social distancing due to the emergence of COVID-19 during that time, we scheduled the interviews online based on preferences and availability. Three researchers were present at each interview to collect data, as the interviews were semistructured. We used the Google Meet platform for the interviews, and most lasted approximately 60 (mean 45, SD 16.26) minutes or more. Each of the 12 participants was interviewed individually, with only participants 5 and 6 attending together as acquaintances. The interviews were conducted in Bengali, audio recorded with the participant’s consent, and we followed the same protocols for both individual and group interviews.

The interviews began with an introduction and a discussion about the participants’ general work experiences during the first wave (March 2020-May 2020) and second wave (March 2021-May 2021) of the COVID-19 pandemic, as this was a new situation for them. We then explored their mental health and the factors that contributed to their stress during both normal and pandemic periods. In addition, we were interested in understanding how the pandemic impacted their social, family, and work lives, as well as what kind of support they received or felt was lacking, which could enhance their overall well-being. Finally, we inquired about any potential technology that could support their well-being. The participants were responsive and willingly shared their perceptions and suggestions.

### Analysis

The sessions were conducted in Bengali and required transcription and translation of all the interviews. Being fluent in both English and Bengali, all authors transcribed and translated the audio carefully directly to English (forward translation). A total of 637 minutes of audio recordings resulted in a rich corpus of narrative data comprising 156 pages (approximately 71,700 words). All authors revised the translated version of the transcription that assured the validity of the data and analyzed it all without using generative artificial intelligence (AI). We created high-level summaries of narrative data for the following interviews. After completing all the interviews, we used reflexive thematic analysis, both inductive and deductive [34,35]. While we had specific topics of interest, such as physicians’ stress, work schedules, sleeping patterns, social problems, and lack of support, we used open inductive coding to identify broader trends [36,37]. We read and reread all the transcriptions multiple times to become thoroughly familiar with the data and subsequently identified 52 different codes across all the transcriptions. We then organized the codes using MIRO, mapping them out, and creating groups of similar codes. We kept in mind the specific topics, and lastly, during theme generation, we adopted a deductive approach that was guided by the model by Daniels et al [31], allowing us to structure our findings within an established theoretical framework. In total, 3 themes were generated.

*Theme 1* focused on the risks and fears that physicians and their families faced regarding the SARS-CoV-2 virus as they worked on the front lines with patients who had undiagnosed infections, which ultimately led to their own infections. *Theme 2* highlighted the increased stress and workload that physicians experienced, indicating that the COVID-19 pandemic negatively impacted their overall well-being. In addition, the social isolation they faced contributed to their mental decline. *Theme 3* addressed the emotional connections that physicians formed with their patients while on duty during the pandemic, as well as the lack of mental support to help them cope with the emotional toll and potential mental breakdowns.

## Results

### Participants Demography

Table 1 shows participants demography, where among the 14 participants, 12 (86%) were female and 2 (14%) were male. We recruited more females for 2 reasons. One, the number of registered female physicians is more (52%) than the male physicians (48%) [38]. Second, women in Bangladesh in general face greater challenges in the workplace due to patriarchal social norms in Bangladesh, which was also the case during the COVID-19 pandemic [38,39]. However, we did not observe any gender-based differences regarding their work experiences during the COVID-19 pandemic (Table 1).

**Table 1:** Participants details (N=14).

Participant code		Marital status	Designation	Format	Unit and type of hospital
**First round of individual interviews**					
	Participant 1	Single	Intern physicians	Videoconferencing	Medicine unit, public hospital
	Participant 2	Single	Medical officer	Videoconferencing	Public hospital
	Participant 3	Married	Medical officer	Videoconferencing	Influenza corner, public hospital
	Participant 4	Single	Medical officer	Videoconferencing	Post–COVID-19 unit, public hospital
**Only group interview**					
	Participant 5	Married	Medical officer	Videoconferencing	Cardiology unit, public hospital
	Participant 6	Single	Doctor, involved in medical research in their own hospitals	Videoconferencing	University medicine department, public university
**Second round of individual interviews**					
	Participant 7	Single	Medical officer	Videoconferencing	Medicine unit, public hospital
	Participant 8	Single	Doctor, involved in medical research in their own hospitals	Videoconferencing	Public hospital
	Participant 9	Single	Intern doctor	Videoconferencing	Public hospital
	Participant 10	Single	Assistant surgeon	Videoconferencing	Private medical hospital
	Participant 11	Single	Intern doctor	Videoconferencing	Medicine unit, public hospital
	Participant 12	Single	Intern doctor	Videoconferencing	Medicine unit, public hospital
	Participant 13	Single	Intern doctor	Videoconferencing	Patriarchal surgery unit, public hospital
	Participant 14	Single	Intern doctor	Videoconferencing	Cardiology unit, private hospital

### Theme 1: COVID-19 Pandemic’s Domino Effect: Undiagnosed Cases Impact Physicians and Their Families Health

#### The Risk From Undiagnosed COVID-19 Cases

During the peak period of the COVID-19 pandemic, the participant physicians primarily attended to undiagnosed patients in emergency zones of hospitals, often unaware of whether these patients were COVID-19 positive, putting the physicians at risk. The situation consisted of cases where patients were unaware of the conditions, and in a few cases, it was deliberate denial.

Many patients did not follow safety precautions while visiting hospitals and were not cautious about their own health conditions. This was due to the high physician-to-patient ratio along with a coping mechanism of handling fear, as a few participants shared. The physicians diagnosed these patients by listening to their concerns and assessing their symptoms, often influenza-like and other respiratory disease symptoms. On the basis of their evaluations, they performed rapid antigen tests to obtain results in about 15 minutes, after which they transferred patients who were suspected to have COVID-19 to the COVID-19 unit for further care. Participant 10 expressed the following:

Most of the time, patients would come to us undiagnosed. They didn’t even know their bodies were carrying the germs of COVID-19 and were unaware of it.

All the physicians noted that patients often concealed their symptoms. This puts the physicians in a difficult position regarding the decision-making of whether to send patients to the COVID-19 pandemic unit. Once in the COVID-19 pandemic unit, patients were generally suspected of having the virus, and due to the lack of personal safety measures, there was a risk of further spread within the hospital. Consequently, physicians suggested that patients return home. As participant 13 explained the following,

We tell them that if they stay in the hospital during the COVID-19 period, they might get infected.

Conversely, when physicians suspected COVID-19 pandemic symptoms—such as breathing difficulties, overlapping infections, or Omicron variant symptoms such as diarrhea and drops in oxygen levels—patients frequently denied they had COVID-19 and only accepted the diagnosis when test results came back positive. This situation increased the risk of physicians getting infected during the assessment period. Participant 6 expressed frustration about the patient’s behavior:

People didn’t admit they had COVID-19. They would say they had stomachaches and wouldn’t mention a sore throat. After examining them, we noticed they had a fever and were coughing. After testing, we found them to be COVID-19 positive. Many of our doctors were affected by these patients.

Some patients had clear signs of COVID-19 but yielded negative test results. As a precaution, physicians often tested themselves 2 to 3 times due to the potential risk of infection. Many physicians reported being infected with COVID-19, yet they continued to work for the well-being of their patients, often sacrificing their own health. Participant 13 shared her experience:

One night, I received seven patients, all suspected to have COVID-19. I also felt sick and distressed, but I didn’t pay much attention and did my duty. It was nearly impossible to maintain proper sterilization and disinfection. I could feel something was happening to me, but I ignored it.

It showed the major difficulty physicians faced, constantly battling the fear inside them.

#### Between Fear and Safety: Families Navigating the COVID-19 Pandemic Threat

The participant physicians often stayed away from families and did not share their concerns around getting infected with the SARS-CoV-2 virus. Several participants were infected with COVID-19 but chose to ignore their conditions due to concern for their families. If they suspected they had contracted the virus, they often refrained from sharing this information, especially with their parents, fearing it would cause unnecessary panic, as participant 1 expressed:

I can’t share these feelings with my family, like telling them I suspect I could be affected. They panic easily. Indeed, people can’t handle panic like a doctor can.

Their primary concern was the fear of transmitting the virus to family members after returning home from work. Physicians who had previously contracted COVID-19 or whose loved ones had experienced it were particularly cautious while on duty, which heightened their tension and fear. Participant 9 noted the following:

Our colleagues who had COVID-19 or whose families suffered heavily due to it took extensive precautions when they returned to the ward. They struggled to breathe, wore N95 masks layered with double surgical masks, and worried about not wanting to be on duty for too long out of fear.

These physicians felt an overwhelming burden regarding the risk their profession posed to their families, often prioritizing their families’ safety over their own, as they shared during the interviews. When family members fell ill, they felt an added responsibility to secure the best medical care for them, which only increased their stress. Some physicians intentionally maintained isolation to minimize interaction with their families to keep them safe. Despite their families expressing concern about their health, these physicians tried alleviating fears. As participant 12 explained the following:

My family used to stress about it. But I wasn’t as fearful because I didn’t expose them to what I was going through. I felt like a carrier, so I lived in the hostel.

This way, the physicians managed to safeguard their loved ones while managing their own health concerns.

#### Physicians’ Struggles of Fighting COVID-19 Along With Families

During the COVID-19 pandemic, the families of physicians also faced significant challenges, as many were infected with the virus. Physicians struggled to balance their hospital duties with their responsibilities at home despite ensuring safety. For instance, participant 3 shared her experience of her baby, aged 14 months, contracting COVID-19 due to her exposure. At the same time, her husband was hospitalized for breathing issues, illustrating the helplessness, stress, and fear she felt regarding her family’s situation, especially as she could not maintain proper isolation with a young child:

Despite maintaining precautions, I was affected by COVID-19 along with my 14-month-old baby; my baby has already suffered because of me. My husband also tested positive and was admitted to the hospital. We couldn’t maintain proper isolation, as my baby was very young then. I wasn’t seriously affected, but the baby was.

Participant 3

During the peak of the COVID-19 outbreak, the patient influx overwhelmed hospitals, pushing them beyond capacity. Therefore, all wards, cabins, emergency units, intensive care units, and critical care units were filled with susceptible patients with COVID-19. This created additional challenges for physicians trying to admit their sick relatives. Participant 6 recounted her struggle to admit her father to the hospital. Meanwhile, her sisters and mother were at home, infected with COVID-19, and their health was deteriorating, adding more to her stress:

I took my father to the hospital and asked for a cabin. There were no empty beds. I called my principal, who advised me to go to another hospital. It took me two hours as a doctor to find a space there; a random person would have needed much longer. My two sisters and my mother were also affected by COVID-19. They couldn’t comprehend how my mother’s oxygen saturation was falling. After three days, I went home, and there was no one else I could ask for help. I eventually took my mother to the cabin where my father was and provided her with oxygen. That night was incredibly stressful.

Participant 6

These experiences reflect a common theme among physicians, showcasing their declining mental and physical well-being as they navigated the dual responsibilities of caring for patients in the hospital and their families at home.

### Theme 2: The Hidden Battles of COVID-19: Physicians’ Burnout, Wellness, and Social Stigmatization

#### Navigating Physicians’ Workload and Wellness

Physicians’ pressures of their professional obligations profoundly impacted their personal lives, leading to multilayered experiences. During the COVID-19 pandemic, the work schedule for health care professionals was severely disrupted for 2 main reasons: an imbalance in the ratio of physicians to patients who were infected compared to normal times and the unavailability of coworkers due to COVID-19 infections. Participant 13 mentioned, “We, four doctors, are treating 50-60 patients simultaneously.” Most patients with COVID-19 were admitted to the medicine unit, significantly increasing the workload. The physicians reported working closely with their senior colleagues to manage the high volume of patients, which led to behavioral changes and occasional outbursts among the staff. Participant 1 shared the following:

In the medicine unit, the mental pressure is greater than that of manual labor. Our seniors have to work even harder, which results in behavioral changes like bad tempers; some even misunderstand each other for no reason and shout.

The rapidly deteriorating conditions of the patients added to the pressure, as physicians frequently had to revisit the same patients while simultaneously attending to new ones. Due to that, many experienced back-to-back morning and night shifts, which were more burdensome than usual. There were instances of 24-hour shifts, and after only a few hours of rest, the physicians had to return to work. This situation was exacerbated by the absence of many coworkers due to COVID-19 pandemic, forcing those present to take on additional responsibilities, negatively affecting their well-being. Participant 11 articulated the complexity of their duties and the resultant stress:

When co-workers skip their duty, we cannot skip ours. If they are assigned 12 patients, we ultimately have to manage their share, which adds pressure on us. It feels especially overwhelming to think about a week of consecutive night duties during that time. Compared to then, duty slots are better now.

These demands greatly impacted their sleep quality. Many physicians experienced difficulties sleeping. Some developed insomnia during the pandemic, which took a long time to resolve. Participant 14 stated the following:

I suffered from insomnia for a while, but establishing a good routine helped me. During that time, I had to take sleeping pills.

This indicates that when they desperately needed sleep, they found it difficult to rest without medication, further disrupting their sleep patterns. Participant 10 shared the following:

I sleep very little at night. I have developed insomnia, so I usually sleep around dawn.

This lack of sleep adversely affected their physical health, leaving them feeling drowsy, with headaches and nausea, which in turn affected their ability to care for patients. Participant 13 remarked the following:

When I don’t sleep enough, I feel drowsy. If my supervisor asks me about a patient’s condition, I stare blankly because I didn’t get adequate sleep; I feel nauseous and have a headache.

This highlighted the lack of support toward the well-being of physicians during the intense period of the pandemic.

#### The Unseen Social Isolation and Stigmatization

Physicians experienced negative social perceptions during the pandemic due to their profession. Many people in their neighborhoods and relatives ignored them, seeing physicians as potential carriers of the virus because of their direct connection with hospitals. This perception took a toll on physicians’ mental health, causing them to feel consistently down.

Many physicians struggled to manage their accommodation during this time because local residents were unwilling to live near them. Participant 3 mentioned that she had to secretly live in a place while hiding her medical laboratory coat:

We found a place through a relative, but we were required to take off our lab coats when returning home so that people wouldn’t know we worked in a COVID-19 dedicated hospital.

Therefore, physicians reported encountering negative attitudes from their neighbors, directly or indirectly. Participant 9 shared that no one in her building knew she was a physician:

Most people didn’t know I am a doctor in my flat. If they knew, they might have treated me like others. I never wore my lab coat while going to work.

Many physicians had to change their living arrangements multiple times. They initially stayed in hotels, but due to costs and safety concerns, they moved to a closed school where they had to sleep on the floor. However, the school committee eventually forced them to relocate. These challenges arose solely because of their work. In even worse situations, relatives refused to let them stay with them, and landlords denied rental applications from physicians. Participant 3 highlighted the mental pressure and uncertainty surrounding these issues:

No relatives would let us stay with them. We struggled to find rentals as landlords immediately refused to rent to us once they knew we were doctors.

Consequently, during Ramadan in the COVID-19 period, the physicians did not have food facilities inside the hospital. So, they had to order food from outside, but they often faced negative attitudes from food delivery riders, who viewed hospitals as risky zones. Therefore, physicians frequently encountered rude behavior from these riders. Participant 6 stated this unhappily:

A rider asked me to come downstairs. I explained that I couldn’t and asked him to come near the gate instead. He rudely replied, “We don’t have any rules for that. COVID-19 is greater in the hospital.” I responded, “Do we produce COVID-19 in medical facilities? If you or your family members were affected by COVID-19, wouldn’t you come here? I have faced such dire situations several times.”

Physicians often faced harassment from traffic police while going out on duty. They had to show their identification to prove they were physicians, but frequently, the police did not believe them and prevented them from reaching their workplaces. The police prioritized enforcing nationwide lockdown restrictions over the physicians’ essential roles in hospitals. Participant 2 expressed her frustration, saying:

When a doctor goes out and shows their ID, the police don’t accept it. They say, “What’s the guarantee that you have a duty now?” It’s social harassment.

This lack of support and social negligence was high during the initial period of the COVID-19 pandemic (March 2020-May 2020).

### Theme 3: Emotional Toll and Lack of Mental Support on Physicians

Physicians often struggle to manage their emotions regarding their patients’ conditions. They experience mixed feelings; they feel joy when patients recover but become stressed and emotionally exhausted when patients worsen. During the pandemic, however, many physicians found it increasingly difficult to cope with their emotions due to the rapid decline and mass deaths of patients. Constant sadness became a regular part of their experience, and some could not handle it. Participant 2 shared a painful memory:

She was 22 years old, with 90% lung problems, and I put her on high-flow oxygen. She had a 1-year-old child who was crying for milk beside her, but I couldn’t stabilize his mother. I felt desperate to save her in any way possible. But eventually, she passed away. It was excruciating for me. Similarly, another patient was admitted two months ago with COVID-19 complications affecting his lungs. His wife was expecting their second baby. We tried desperately to save him, but suddenly, he passed away. These experiences affected us emotionally a lot.

When physicians treat patients for an extended period, they often develop strong emotional attachments, especially to those older or similar in age. Patients can feel like family to them. The physicians invest a great deal of effort into trying to improve their patients’ conditions, and when they are unable to do so, they experience significant emotional experiences. Participant 6 noted the following:

I stayed in a ward for so long that there was an attachment. It felt terrible seeing 28- to 30-year-old boys and girls come into the ward with COVID-19 and then witnessing their drastic decline. I couldn’t do anything.

Declaring a patient’s death adds another layer of stress for physicians, as it involves both emotional and professional repercussions, particularly when informing the patient’s family. Physicians experience immense psychological strain as they repeatedly witness their patients die. Participant 1 expressed her feelings:

The issue of declaring death causes my heart to race irregularly. One night shift, I was sad and had to declare four deaths in one night. I couldn’t sleep that night; it was my most stressful night.

Despite these overwhelming situations, many physicians reported a lack of mental support or counseling to help them manage the emotional toll. They often resort to self-counseling to cope, while in many Western countries, physicians’ mental health is routinely prioritized. Participant 5 highlighted this gap in the Bangladeshi medical system, where taking a leave to focus on mental health is nearly impossible:

There is no counseling system. You have to continue your duties and manage everything on your own. Even if you must take leave for your mental well-being, you must find a replacement doctor to assume your duties. No matter what, you cannot leave the hospital ward empty. A co-worker of mine struggled greatly when her parents were hospitalized with COVID-19, but she couldn’t take leave and had to fulfill her responsibilities.

Physicians also encountered significant limitations in providing counseling services to patients’ families during COVID-19. The sudden, tragic deaths of patients, who were often otherwise healthy, left families overwhelmed. Participant 2 commented the following:

Counseling services are limited in our country. When a patient passes away, their family members often don’t want to hear anything. It’s tough. Sometimes, one family member blames another for their own mental satisfaction.

This underscores the need for counseling sessions to support mental health among all stakeholders within the health care system. Participant 14 suggested the following:

It would be very helpful if we had a group where doctors could share their problems and consult each other. Learning from others’ experiences would help us mentally prepare for challenging cases and provide comfort in difficult situations.

## Discussion

### Principal Findings

Our results highlighted the struggles faced by Bangladeshi physicians and the decline in their physical and mental well-being during the COVID-19 pandemic. These physicians had to attend to an excessive number of patients, often in close physical contact, exposing themselves to the risk of infection. This heightened their anxiety, especially with fears of transmitting the virus to their families. Many physicians, along with their family members, contracted COVID-19, which hindered their ability to provide adequate support at home due to the widespread crisis. In some cases, physicians chose not to reveal their COVID-19 symptoms to reduce fear and panic among their family members. As they worked tirelessly to manage a high volume of patients, they often could not take necessary breaks, even when unwell. This overwhelming workload compromised their physical health. Moreover, instead of receiving societal support, many physicians faced negative attitudes from the public, which further impacted their mental health. The loss of patients also took an emotional toll on these health care professionals. While counseling could help them cope with these challenges, the lack of a nationwide counseling system leaves many physicians feeling stressed and burned out.

To support physicians’ mental and physical well-being, we need to consider well-accepted contextual support interventions that involve physicians and other stakeholders in society. During the pandemic, Daniels et al [31] proposed a grounded and coherent model based on the experiences and psychological care needs of frontline COVID-19 physicians in the United Kingdom, derived from ethnographic research. This credible model inspired us to develop our own contextual model, incorporating similar components while expanding to include available technological solutions for supporting physicians’ health.

### The Pyramid Model

**Figure 1:**
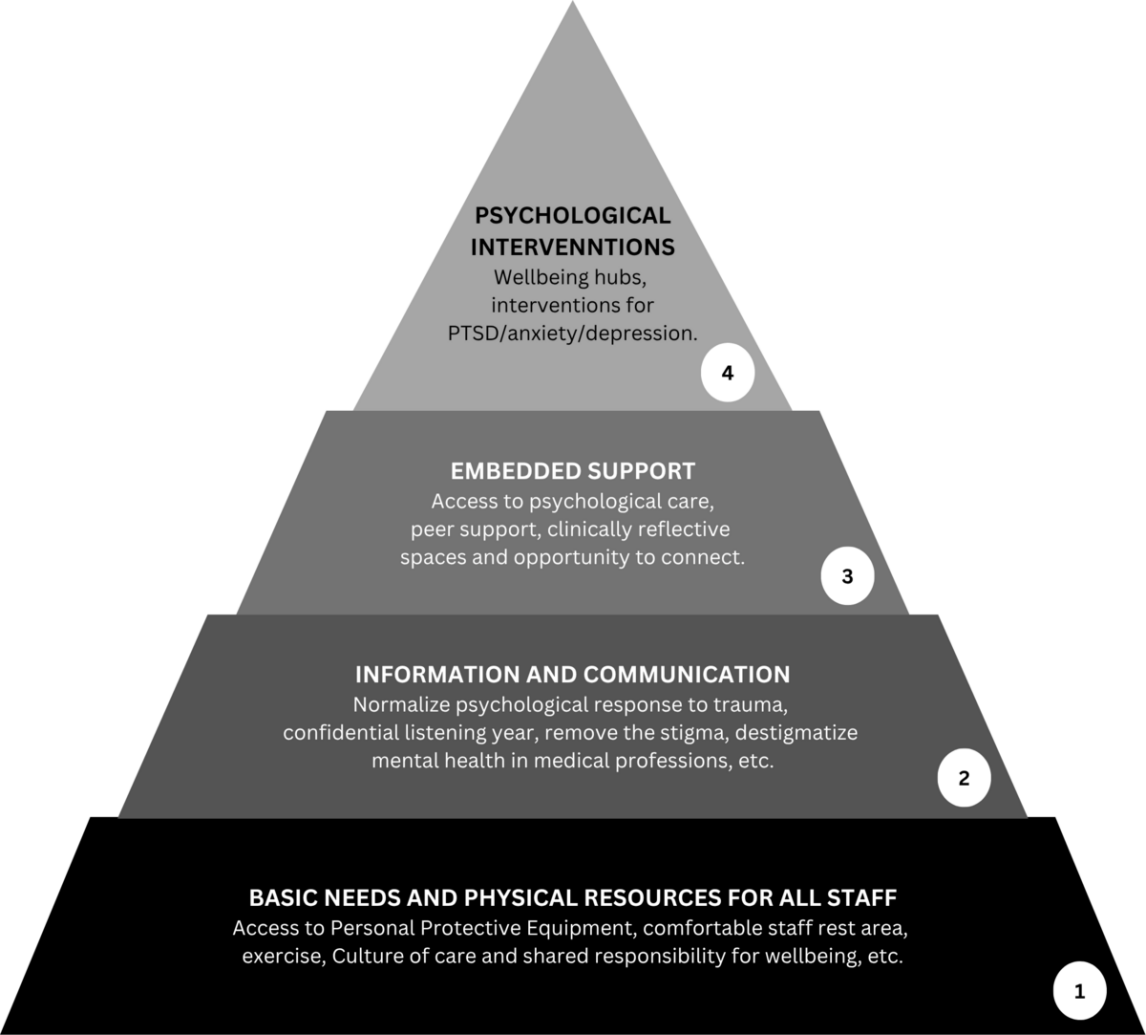
Model of well-being and psychological care for frontline physicians (reproduced from Daniels et al [31], which is published under Creative Commons Attribution 4.0 International License [40]). PTSD: posttraumatic stress disorder.

The pyramid bottom-up model by Daniels et al [31] of well-being and psychological care for physicians consists of four layers: (1) basic needs and physiological resources, (2) information and communication, (3) embedded support, and (4) psychological interventions, as illustrated in Figure 1.

The first layer emphasized the importance of implementing local guidelines for proper nutrition, rest, and sleep. They suggested creating designated, quiet, confidential spaces for the physicians and the staff to reflect on and seek support. Fostering a culture of care and shared responsibility for staff well-being is essential, with senior leaders playing a key role in modeling positive behaviors related to well-being. In addition, they highlighted the need for personal safety measures.

The second layer focused on the fact that stressful and traumatic incidents require open discussions on their psychological effects and the promotion of mental health awareness in medical training. Staff should be educated on warning signs of mental health deterioration, such as emotional responses and absenteeism, and should have a safe space to discuss their well-being without fear of negative repercussions.

The third layer emphasized that all physicians should have access to integrated psychological support. This includes face-to-face or video call reflective sessions among peers and senior staff within clinical teams. In addition, structured peer-to-peer interventions, such as Trauma Risk Management or the “StartWell>EndWell” approach, should be readily available for enhanced support.

Finally, in the last layer, psychological interventions should be tailored to individual needs while considering team well-being and available in group, personal, and online formats. It should be able to manage the impact of fear, anxiety, panic, guilt, hopelessness, disconnection, and compassion fatigue among health care workers. Support should address issues such as moral injury, trauma, posttraumatic stress disorder, depression, and mental health stigma.

### Comparison Analysis

The research by Daniels et al [31] shows physicians often cope with stress through various strategies, such as exercise, spending time outdoors, expressing their feelings, practicing mindfulness, and journaling, which is absent for Bangladeshi physicians. However, many reported storing emotions due to limited time to process events, leading to emotional disconnection, particularly when dealing with patients who are severely ill or dying. Bangladeshi physicians also cannot vent out their emotions. Physicians in the United Kingdom often prioritized others’ needs over their own, setting aside personal well-being to support colleagues or patients, which a few physicians in Bangladesh also do. The research by Daniels et al [31] shows support systems included talking to coworkers, accessing embedded psychological services, formal therapy, mental health apps, phone support, and messaging groups, as well as relying on friends and family. Barriers to support included lack of time for discussions or attending sessions. Workplace wellness initiatives were mentioned, with small acts of kindness or environmental adjustments having a notable positive impact. Negative influences included poor communication, ineffective leadership, and government decisions. However, in Bangladesh, no support system exists for the physicians to assist them in their well-being. Hence, an improvement in the system is required along with a supportive network. This motivates us to work for the contextual support system, influenced by the research model by Daniels et al [31].

### Proposed Trajectile Model for Physicians’ Well-Being

#### Overview

**Figure 2:**
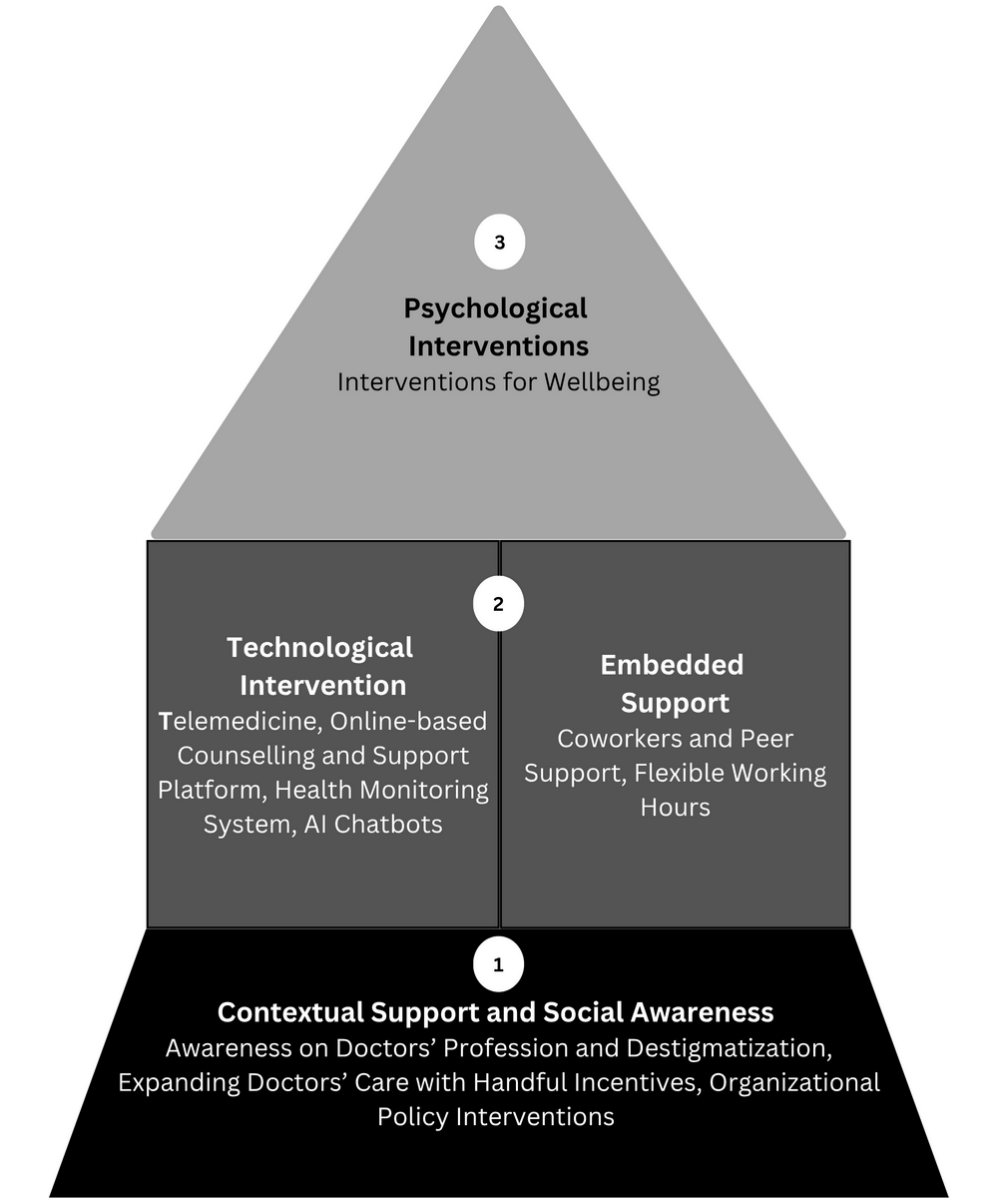
Proposed trajectile model for physicians’ well-being. AI: artificial intelligence.

Our trajectile bottom-up model for physicians’ well-being is depicted in 3 layers, shown in Figure 2. We built upon our findings to develop this model and incorporated contextual and technological components, including AI interventions. The layers of our model include (1) contextual support and social awareness, (2) technological interventions and embedded support, and (3) psychological interventions. We aligned our findings with the proposed model by Daniels et al [31] (Figure 1) and then modified and integrated components to create our proposed model (Figure 2). For example, the embedded support and psychological intervention layers are similar to the model by Daniels et al [31]. We recommend contextual support and social awareness, and the technological interventions components in the trajectile model differ from the model by Daniels et al [31], but that inspired us. The proposed model can be a sustainable model with effective implementation and can be adaptable in any situation and culture.

#### Layer 1: Contextual Support and Social Awareness

##### Overview

In the first layer of the trajectile models, we recommend enhancing contextual support and social awareness for physicians. This includes raising awareness about the medical profession, reducing stigma, expanding support for physicians through various incentives, and implementing policy interventions aimed at promoting both their mental and physical well-being.

##### Awareness of Physicians’ Profession and Destigmatization

We found that physicians often faced negative attitudes and stigma from society during the COVID-19 pandemic, which can lead to feelings of stress. In addition, physicians tend to refrain from expressing their emotions in the workplace because they fear others might interpret their feelings as signs of fear or guilt rather than understanding that they genuinely care for their patients (referring to themes 2 and 3). In Bangladesh, for instance, many people blame physicians for patient losses due to perceived negligence [41]. Accordingly, Galbraith et al [13] highlighted methods for destigmatizing the workplace for physicians, such as fostering an open communication culture, organizing team-building activities, and providing peer support training during health crises and traumatic events, which can include nonmedical personnel [42,43].

However, in our context, most stigmatization occurs at the societal level. It is crucial for society to understand the challenges and hardships that physicians face and to offer expanded support accordingly. Key strategies for preventing blame and psychological trauma among physicians include raising public awareness, allowing physicians to maintain a sense of self-determination, and encouraging effective communication skills [28]. By implementing these strategies with the stakeholders around physicians, society can better appreciate physicians’ dedication to their patient care.

##### Expanding Physicians’ Care With Handful Incentives

Our physicians have mentioned that they often feel outnumbered by the increasing number of patients during the pandemic (referring to theme 2). This situation highlights the need to increase the number of physicians [44-47]. A significant reason is that rural patients tend to migrate to cities because there are fewer physicians in rural areas. In addition, many physicians are reluctant to work in these regions due to logistical challenges, such as low incentives, limited medical support, and inadequate accommodation options [48]. Previous research has also indicated that physicians do not receive sufficient recognition through incentives [21,28,44,47]. Bangladesh faces a similar issue, with only 5.26 physicians available for every 10,000 people [48]. Furthermore, their incentives are minimal, often no more than US $270, largely due to bureaucratic obstacles [49]. To address the shortage of physicians, the government should expand medical education and specialized training programs to ensure that more physicians are prepared to serve the rural population effectively. Establishing policies that provide adequate incentives in these areas is also essential. Many countries have already adopted performance-based incentive programs alongside salaries, and evidence shows that such incentives can significantly improve health care providers’ performance [50-52]. By implementing these measures, we can develop a nationwide health care system that is more evenly distributed and sustainable. This system will help alleviate the patient overload in urban areas while extending vital medical support to rural regions.

##### Organizational Policy Interventions

Physicians often work irregular hours, which negatively impacts their well-being (referring to theme 2). To address this issue, hospital administration must revise policies to support physicians’ health for the greater good. Certain measures, such as providing personal space for rest, protecting against occupational hazards, offering childcare opportunities, and implementing incentives and maternity and parental leave, can help prevent burnout among health care workers [31,53]. However, many public hospitals in Bangladesh lack these provisions, while some private hospitals offer them only to a limited number of physicians. Therefore, hospital administration should implement these measures within the health care system so the government can take the necessary steps to mandate these policies.

#### Layer 2: Technological Intervention and Embedded Support

In this second layer, we included 2 components: technological interventions (such as telemedicine, online support platforms, health monitoring systems, and AI chatbots) and embedded support (such as coworkers and peer support and flexible working hours), essential for improving physicians’ health.

##### Technological Intervention

###### Telemedicine

Our physicians have expressed concerns about the risk of COVID-19 infection from undiagnosed patients and the necessity of physical contact (referring to theme 1). Telemedicine is crucial in reducing physical interactions between physicians and patients, thereby minimizing the risk of exposure to various viruses and diseases, including the SARS-CoV-2 [54]. Physicians can provide clinical care through telephone and video consultations, especially since COVID-19 significantly affects older populations [55].

Bangladesh offered several telemedicine services during the pandemic, such as “Tonic” and “ShebaGhor.” However, these services struggled to sustain themselves due to inadequate regulations, patient trust, and limited resources [54]. In addition, many people in Bangladesh are not accustomed to telemedicine and face challenges related to access to information and communication technology, leading them to prefer in-person consultations. Nevertheless, we advocate for the adoption of telemedicine, as general practice in the United Kingdom has successfully transitioned to telephone or video consultations. This shift has reduced unnecessary face-to-face interactions and the associated risk of virus transmission, easing concerns about contracting illnesses [56].

The effectiveness of telemedicine is evident in high-income nations, where the system is regulated by the government. In contrast, telemedicine services in Bangladesh operate independently, raising public trust issues. The Bangladesh Medical and Dental Council should establish proper regulations to create a sustainable telemedicine system. Hospitals could then operate toll-free telemedicine hotlines to broaden services and enhance accessibility. A single toll-free number could connect callers to local hospitals. Bangladesh already has emergency service numbers in place; using and expanding this existing infrastructure could help address the needs of a large patient population and benefit physicians’ health.

###### Online Counseling and Support Platform

Physicians often seek counseling to express their emotions, but they face a lack of options, particularly in resource-limited countries such as Bangladesh, where hospitals do not have proper counseling units (referring to theme 3). Digital platforms can support physicians’ mental health by providing easy access to counseling services, peer connections, and stress management tools. These resources can help them navigate the challenges of their profession and alleviate stress. Digital and technological platforms supplement traditional behavioral support [57]. Counseling delivered over the phone or through the internet can effectively manage physicians’ mental health [58], but it should be easily available and accessible and timely [59,60]. To address this need, the Bangladesh Medical and Dental Council could establish an online counseling platform accessible only to physicians, enabling them to receive the support they require from anywhere.

###### Health Monitoring System

We additionally recommend technology-based health monitoring systems that can assist physicians in tracking their health and mental well-being. As participants are lacking in maintaining their personal well-being at this point, cost-effective wearable devices, such as Fitbit (Google LLC), offer valuable insights into various health metrics and self-assessment. For instance, Fitbit can monitor sleep patterns, physical activity, blood oxygen levels, heart rate variability, and exercise frequency, all of which can indicate a person’s healthy lifestyle [61] and stress levels. This enables physicians to support their own health and pursue a healthier lifestyle. In addition, Anmella et al [62] developed 2 chatbots designed to screen, monitor, and mitigate anxiety and depressive symptoms as well as work-related burnout while also assessing suicide risk in physicians through conversational interactions. Such technology can help identify stress among physicians and alert relevant authorities or family members, enabling them to support the physicians’ well-being. Meanwhile, physicians can use these tools to engage in self-care and self-medication therapies.

###### AI Chatbots

There is often a significant lack of resources in resource-limited countries, leading to accessibility issues for counseling tools and digital platforms. Participants also explicitly mentioned their lack of counseling support (referring to theme 3). AI chatbots can provide a cost-effective solution for resource-limited countries such as Bangladesh. Baek et al [63] suggested that AI chatbots can help improve states of anxiety, depression, and burnout. For instance, the Woebot chatbot has assisted young adults with depression and anxiety, resulting in a significant reduction in both conditions [64]. Similarly, the Vivibot chatbot contributed to reducing anxiety, although it did not have an effect on depression [65]. Today, AI technologies, such as ChatGPT by OpenAI, Gemini by Google LLC, Gork by X, and Meta from Meta are widely accessible and can offer open-ended well-being suggestions. However, these systems often lack personalization, making it difficult to address specific mental health issues effectively. Designing a mobile app that combines existing chatbots while incorporating personalized features is feasible. Therefore, AI chatbots could serve as a valuable tool to support the psychological well-being of physicians by providing personalized and accessible interventions, especially in resource-limited countries.

##### Embedded Support

###### Coworkers and Peer Support

We observed that when coworkers fall ill and request assistance, they must address those issues immediately, increasing pressure during the pandemic (referring to theme 2). On the other hand, coworkers also supported one another during their illnesses. This support is essential in the medical field to maintain workplace efficiency and the mental well-being of physicians. Having reliable, responsible, empathetic, accountable, and supportive coworkers during the pandemic was invaluable for navigating challenges. Senior coworkers provided clear guidelines, served as role models for junior physicians, and fostered a culture of learning and collaboration among peers [22]. This support is also considered peer support, which can be extended to social support with regular contact via phone call or video chat with colleagues, family, and friends [57]. Therefore, these supportive relationships can significantly enhance one another’s well-being.

###### Flexible Working Hours

Physicians have said that they often work long hours without any breaks, leading to mental exhaustion (referring to theme 2). They should be given a flexible schedule with adequate breaks between shifts to address this issue. In Bangladesh, interns experience unstructured duty hours; most physicians work daily. Urooj et al [22] suggest that minimizing physicians’ duty hours and limiting routine checkups can improve their health and reduce the number of staff working at any given time, allowing some to be reserved as backups. This approach would help achieve a better work-life balance [53]. Therefore, the administration needs to redefine and structure physicians’ working hours, and the Bangladesh Medical and Dental Council should implement a policy to support these changes. This would ensure that the support provided is appropriately fixed and effective.

#### Layer 3: Psychological Interventions

We refer to psychological interventions similar to the model proposed by Daniels et al [31], which focuses on addressing individuals’ needs while managing the effects of anxiety, hopelessness, and stress. The physicians involved in the study expressed a desire for regular monthly counseling sessions due to the significant loss of patients during every crisis. Therefore, counselors and volunteers could play a crucial role in not only addressing acute symptoms, such as depression, anxiety, and psychological distress, but also in supporting physicians during the postpandemic phase and helping them cope with ongoing challenges. This support intervention should be available online and in hospitals with easy access.

### Limitations and the Way Forward

We interviewed 14 participants online during the pandemic, all from the Chattogram region. This region is around 300 km southeast of the capital but is an overpopulated city. The medical support system was weaker than the capital’s. We were able to access these physicians during this emergency period. Although we aimed to expand our interviews to different cities, such as the capital Dhaka, so that we could perform comparative analysis, many potential participants did not respond in Dhaka. This presents a limitation, as the findings may not be generalizable. However, they reflect physicians’ overall experiences during the pandemic, which aligns with the situation of most physicians who worked during the COVID-19 pandemic in Bangladesh. In addition, the proposed model was not tested or validated. Testing the model with the stakeholders may give us valuable insight into its acceptability and usability.

Future research can be expanded by, including physicians and stakeholders from hospitals and the Bangladesh Medical and Dental Council, as many institutional issues are linked to hospital administration and policy makers within the council. Engaging these groups in further studies will provide insights into effective interventions for maintaining physicians’ well-being. In addition, we plan to conduct longitudinal studies using wearable technologies, such as Fitbits, to gather real-time physiological data from physicians. Analyzing these data will help identify stress factors associated with their work, which may contribute to redesigning their work environment in the future.

### Conclusions

A qualitative study involving 14 physicians explored their experiences and well-being during the COVID-19 pandemic. This study helped us identify their challenges in maintaining their well-being amid various contextual difficulties. We recognized the importance of addressing these concerns and set out to develop a contextual intervention model. Our model for promoting well-being was inspired by the well-being framework by Daniels et al [31], incorporating contextual and technological interventions. We proposed suggestions based on a bottom-up approach, highlighting interconnected concepts. By focusing on this model, we aim to engage stakeholders in rethinking and redesigning the work environment for physicians. This approach is designed to be sustainable over the long term and adaptable to any situation and culture.

## Abbreviations

AI: artificial intelligence
